# Severe neonatal multiple sulfatase deficiency presenting with hydrops fetalis in a preterm birth patient

**DOI:** 10.1002/jmd2.12074

**Published:** 2019-08-20

**Authors:** Lars Schlotawa, Thomas Dierks, Sophie Christoph, Eva Cloppenburg, Andreas Ohlenbusch, G. Christoph Korenke, Jutta Gärtner

**Affiliations:** ^1^ Department of Paediatrics and Adolescent Medicine University Medical Center Göttingen Göttingen Germany; ^2^ Department of Chemistry, Biochemistry I Bielefeld University Bielefeld Germany; ^3^ Department of Child Neurology and Metabolic Disorders, Medical Centre Oldenburg University Children's Hospital Oldenburg Oldenburg Germany; ^4^ Department of Neonatology, Intensive Care Medicine and Paediatric Cardiology, Medical Centre Oldenburg University Children's Hospital Oldenburg Oldenburg Germany

**Keywords:** formylglycine generating enzyme, hydrops fetalis, lysosomal storage disorder, multiple sulfatase deficiency, preterm birth, *SUMF1*

## Abstract

Multiple sulfatase deficiency (MSD) is an ultra‐rare lysosomal storage disorder (LSD). Mutations in the *SUMF1* gene encoding the formylglycine generating enzyme (FGE) result in an unstable FGE protein with reduced enzymatic activity, thereby affecting the posttranslational activation of newly synthesized sulfatases. Complete absence of FGE function results in the most severe clinical form of MSD with neonatal onset and rapid deterioration. We report on a preterm infant presenting with hydrops fetalis, lung hypoplasia, and dysmorphism as major clinical signs. The patient died after 6 days from an intraventricular hemorrhage followed by multi‐organ failure. MSD was caused by a homozygous *SUMF1* stop mutation (c.191C>A, p.Ser64Ter). FGE protein and sulfatase activities were absent in patient fibroblasts. Hydrops fetalis is a rare symptom of LSDs and should be considered in the differential diagnosis in combination with dysmorphism. The diagnostic set up should include measurements of glycosaminoglycan excretion and lysosomal enzyme activities, among them at least two sulfatases, and molecular confirmation.

**Abbreviations**
FGE: formylglycine generating enzyme
LSD: lysosomal storage disorder
MSD: multiple sulfatase deficiency
NIHF: nonimmunological hydrops fetalis
NVS: neonatal very severe MSD
SNP: single nucleotide polymorphism
*SUMF1*: sulfatase modifying factor 1

## 1 INTRODUCTION

Multiple sulfatase deficiency (MSD, MIM #272200) is a rare lysosomal storage disorder (LSD) caused by the combined deficiency of cellular sulfatases resulting in a complex LSD. Clinical features comprise variable signs of developmental delay followed by progressive loss of motor and cognitive function, dysmorphism, organomegaly and an ichthyotic skin rash.^1^ MSD is caused by mutations in the sulfatase modifying factor 1 gene (*SUMF1*) resulting in misfolding and early degradation of catalytically impaired formylglycine generating enzyme (FGE).^2^ FGE activates newly synthesized sulfatases through the oxidation of a conserved cysteine to formylglycine, which is the essential catalytic residue of every sulfatase (17 nonredundant enzymes in humans). FGE malfunctioning results in absent or reduced sulfatase activities in different cellular compartments causing the unique combination of clinical features from single sulfatase deficiencies in MSD.^3^, ^4^ More than 50 different *SUMF1* mutations have been described, most of them missense mutations. Different forms of MSD can be distinguished based on time of onset and clinical severity that are determined by residual functionality of FGE variants.^5^ Mutations generating less severe FGE dysfunction were found in attenuated cases, whereas deletions, frameshift, or early stop mutations result in complete protein loss and severe forms of MSD (neonatal very severe MSD, NVS).^2^ Only few of such NVS patients have been published before.^2^, ^6^, ^7^, ^8^, ^9^, ^10^

Hydrops fetalis is a severe pregnancy condition. Whereas immunological hydrops fetalis caused by fetal anemia due to rhesus incompatibility has historically been the most frequent cause, nonimmunological disorders cause more than 85% of hydrops fetalis nowadays with an incidence of 1 in 2000‐3000 pregnancies.^11^, ^12^ Actual cases result from infectious diseases, congenital heart defects, feto‐fetal transfusion, and genetic diseases.^11^, ^13^ Inborn errors of metabolism are rare causes of nonimmunological hydrops fetalis (NIHF), with LSDs being more common than others. NHIF is a symptom in about 14 different LSDs.^14^, ^15^ While various pathophysiological processes have been discussed to favor NIHF, the genuine pathophysiology remains unknown.^16^, ^17^

Here we describe pathological findings in a preterm neonate presenting with a hydrops fetalis as leading symptom in a neonatal very severe and rarely seen form of MSD.

## 2 MATERIALS AND METHODS

Cell culture, DNA, RNA, protein extraction, sequencing of the *SUMF1* gene, sulfatase activity assays, and FGE western blot was performed as described before.^2^ We used a polyclonal rabbit anti‐transferrin antibody, concentration 1:10 000 in PBS/5% nonfat dried milk, as loading control (DakoCytomation, Glostrup, Denmark, Cat. No. A0061).

## 3 PATIENT REPORT

The male patient is the third child of a nonconsanguineous German couple. Birth weight 2000 g (71st centile), length 40 cm (19th centile), and head circumference 31 cm (67th centile). The mother had a history of two previous miscarriages and two elder siblings are healthy. From 19 weeks of gestation, scans showed a significant ascites in the fetus. The baby was born at 31 + 5 weeks via caesarean section after premature rupture of membranes.

The patient showed a flattened nose, epicanthal folds, short limbs, a small chest, and a wide abdomen. The postnatal abdominal ultrasound showed a massive ascites that required drainage. A persistent fetal circulation was detected as well as reduced right and left ventricular output. Chest X‐ray revealed respiratory distress syndrome and bilateral lung hypoplasia. Dilated cerebral ventricles were seen on cranial ultrasound scan. Excess excretion of chondroitin sulfate and dermatan sulfate was present in urine samples.

The patient required intubation followed by continuous ventilation starting directly after birth. Blood circulation and low blood pressure required immediate therapy with inotropes. An intraventricular hemorrhage was noted on day 3 complicated by an intraparenchymal bleeding and signs of intracranial pressure. The patient died on day 6 because of multi‐organ failure.

## 4 RESULTS

**Figure 1:**
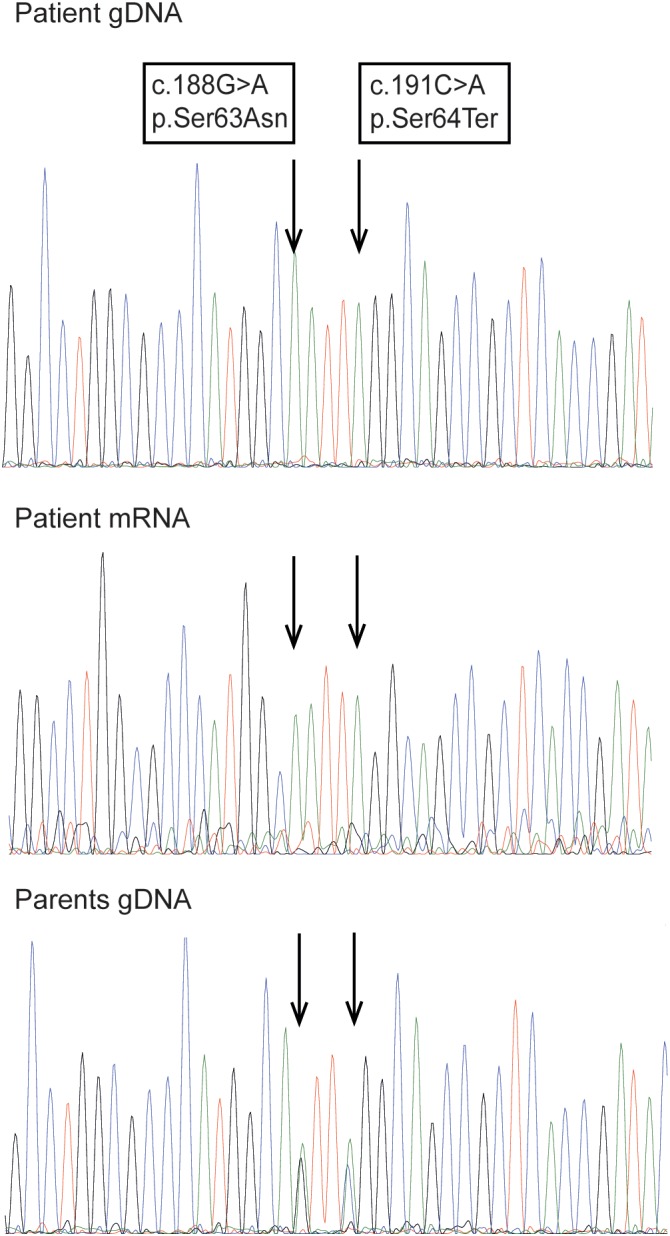
Molecular analysis of *SUMF1*. Sequences of the *SUMF1* gene in genomic DNA of the patient and both parents, and in mRNA of the patient isolated from patient fibroblasts. The analysis showed a homozygous mutation c.191C>A, p.Ser64Ter on gDNA and mRNA levels for the patient and the same mutation in heterozygosity in the parents. In addition, the patients' gDNA and mRNA carried the described SNP c.188G>A, p.Ser63Asn that was found in heterozygosity in the parents' gDNA

The activities of four different lysosomal sulfatases and nonlysosomal steroidsulfatase were absent or drastically reduced in patient's fibroblasts. Other lysosomal hydrolases showed normal activities (Table 1). Sequencing of *SUMF1* revealed a homozygous mutation c.191C>A creating a TAG stop codon at position 64 (p.Ser64Ter) in the FGE amino acid sequence. Both parents were heterozygous for the mutation. *SUMF1* mRNA was fully transcribed in patient fibroblasts, although at reduced levels. In addition, child and parents carried the previously reported benign sequence variant c.188G>A, p.Ser63Asn (Figure 1).

**Table 1:** Activities of sulfatases and control lysosomal hydrolases in patient fibroblasts

Enzyme	Activity (nmol/h per mg)	Reference range (nmol/h per mg)
Arylsulfatase A	9	387‐1093
Arylsulfatase B	0	177‐547
Steroidsulfatase	1^a^	55‐127^a^
Iduronate‐2‐sulfatase	0	3‐20
*N*‐Acetylgalactosamine‐6‐sulfatase	0	6‐9
α‐l‐Iduronidase	181	34‐139
β‐Hexosaminidase A + B	8375	1806‐12 942

a (pmol/h per mg).

**Figure 2:**
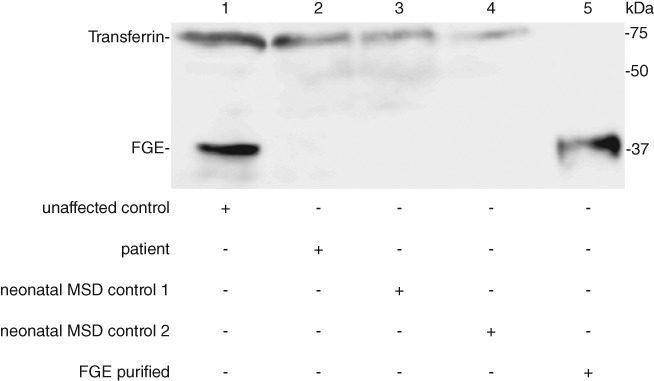
FGE expression analysis. Western blot analysis of FGE expression in lysates from patient's fibroblasts in comparison to unaffected control fibroblasts and two different neonatal MSD fibroblast lines, endogenous transferrin and purified FGE served as loading controls (n = 3). No FGE expression could be detected in the patient's sample and the neonatal MSD cell lines. FGE, formylglycine generating enzyme; MSD, multiple sulfatase deficiency

No FGE was detected by western blot analysis in lysates of patient's fibroblasts. Fibroblast lysates from two other neonatal very severe MSD cases served as controls and did not show detectable FGE expression, whereas FGE was clearly detectable in lysates of non‐MSD fibroblasts (Figure 2).

## 5 DISCUSSION

The dysmorphic features in the patient and increased urinary GAG excretion were suspicious for a mucopolysaccharidosis. LSDs, including different forms of mucopolysaccharidosis, are a rare cause of NIHF and MSD is one of the rarest entities.^1^, ^15^ The majority of MSD cases are of the late infantile type, only few neonatal cases have been described. Some patients presented with hydrops fetalis but all shared an eventful neonatal period with great likelihood of early death. All affected MSD newborns had symptoms in common that resemble mucopolysaccharidosis such as coarse facial appearance, corneal clouding, hydrocephalus and skeletal changes. Ichthyotic skin rash, one of the leading clinical sign in MSD with later onset, is not regularly detected in neonatal cases.^2^, ^6^, ^7^, ^8^, ^9^, ^10^ A predominantly neurological deterioration and developmental delay or absent psychomotor development appears only in MSD cases with later onset.^1^, ^18^

This report describes the earliest onset of MSD to date. Dysmorphic features and hydrops fetalis, present since the 19th week of gestation, seem to be the earliest clinical signs in neonatal MSD. The cardiac problems as well as the lung hypoplasia are likely an effect of hydrops fetalis but cannot be excluded as a genuine symptom of MSD.

Sulfatase activities in the patient were absent or below the detection limit (Table 1). Complete absence of sulfatase activities in MSD is considered to cause the severest MSD phenotype.^2^ In all NVS cases described so far residual sulfatase activities were either absent or extremely reduced like in our patient. This is supposed to result from absent FGE activity caused by a complete loss of function due to nonsense mutations or severe missense mutations.^2^, ^6^, ^7^, ^8^, ^9^, ^10^ Of note, also the *Sumf1* gene‐trap MSD mouse model showed absent sulfatase activities in all tested tissues and displayed a severe phenotype with early lethality, growth retardation, bone changes and dysmorphic features.^19^ On the other hand, hypomorph SUMF1 variants represent the majority of mutations found in MSD. They destabilize FGE but allow for some residual activity. In line with this, sulfatases also show residual activities and such MSD patients present with late infantile or juvenile MSD types.^2^, ^5^

The homozygous mutation detected in the present patient results in a complete loss of FGE function. No FGE protein could be detected in lysates from patient fibroblasts. Endogenous mRNA was detectable, but only at low amounts likely as a result of nonsense mediated decay. Due to homozygosity, all clinical findings in our patient are a direct result of a single mutation. The *SUMF1* c.191C>A, p.Ser64Ter mutation is one of the most deleterious in MSD. The same mutation was published in compound heterozygosity in combination with an undescribed missense mutation in a patient that displayed neonatal dysmorphic features but appeared to be less severely affected than our patient and other NVS MSD cases.^8^ This is most likely a result of residual FGE activity of the second, putative hypomorph mutation in this child and less likely the influence of yet unknown disease modifiers in MSD. A genotype‐phenotype correlation in NVS MSD cases based on different SUMF1 nonsense mutations remains speculative until more cases with a defined genotype will be described.

Until today, MSD is untreatable. We strongly recommend considering hydrops fetalis as an early clinical sign of LSDs and MSD.^20^ Enzymatic and genetic testing for this group of diseases with special emphasis on sulfatase function should be part of a routine diagnostic set up after the detection of hydrops fetalis in the unborn or newborn child.

## CONFLICT OF INTEREST

Lars Schlotawa, Thomas Dierks, Sophie Christoph, Eva Cloppenburg, Andreas Ohlenbusch, Korenke Cristoph, and Jutta Gärtner declare no conflict of interest.

## INFORMED CONSENT

All procedures followed were in accordance with the ethical standards of the responsible committee on human experimentation (institutional and national) and with the Helsinki Declaration of 1975, as revised in 2000(5). Additional informed consent was obtained from the patients parents for being included in this case report.

## Supporting information


**Figure S1** Original file of the FGE expression Western blot used for Figure 2.
