# Supplementary figures and images for: Severe neonatal multiple sulfatase deficiency presenting with hydrops fetalis in a preterm birth patient
Source: JIMD Rep. 2019 Aug 20;49(1):48–52. doi: 10.1002/jmd2.12074 (PMC6718111; doi:10.1002/jmd2.12074)

**Supplementary Figure 1**

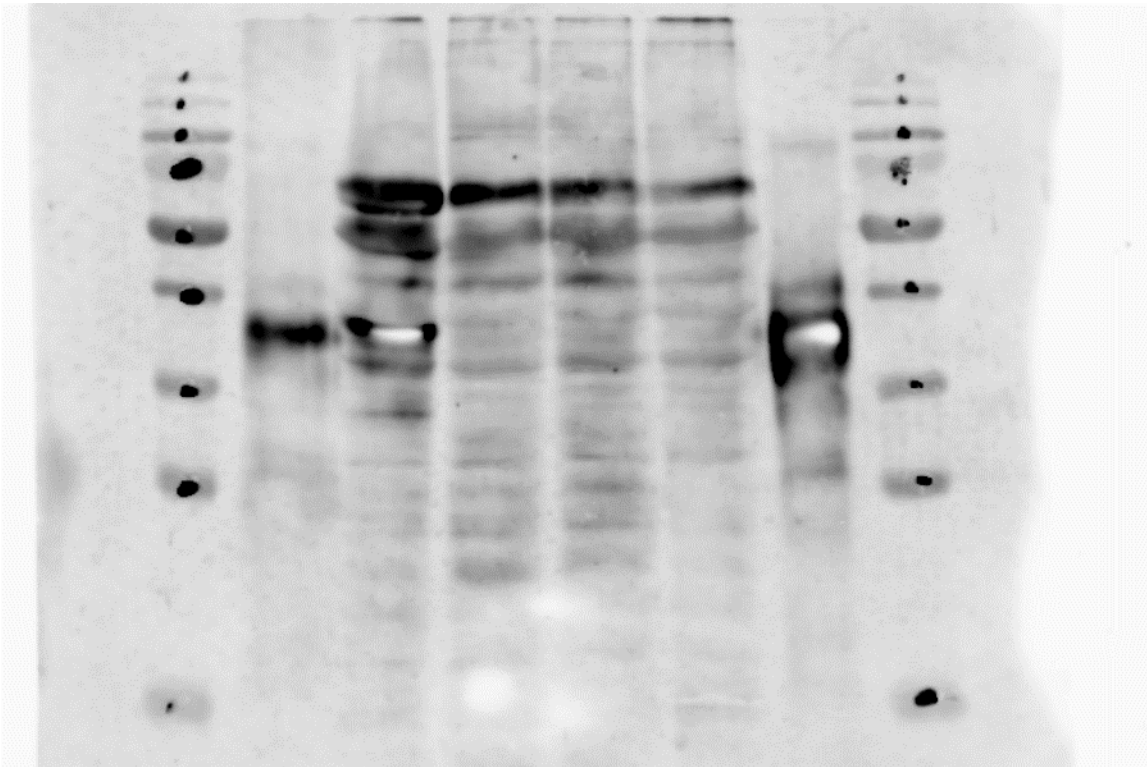

Supplement: Supplementary file 1 — Figure S1 Original file of the FGE expression Western blot used for Figure 2. [file JMD2-49-48-s001.pdf]
